# Communicative Blame in Online Communication of the COVID-19 Pandemic: Computational Approach of Stigmatizing Cues and Negative Sentiment Gauged With Automated Analytic Techniques

**DOI:** 10.2196/21504

**Published:** 2020-11-25

**Authors:** Angela Chang, Peter Johannes Schulz, ShengTsung Tu, Matthew Tingchi Liu

**Affiliations:** 1 Department of Communication Faculty of Social Sciences University of Macau Macao China; 2 Institute of Communication and Health Lugano University Lugano Switzerland; 3 Department of Radio and Television Ming Chuan University Taipei Taiwan; 4 Department of Management and Marketing Faculty of Business Administration University of Macau Macao China

**Keywords:** placing blame, culprits, sentiment analysis, infodemic analysis, political grievances, COVID-19, communication, pandemic, social media, negativity, infodemic, infodemiology, infoveillance, blame, stigma

## Abstract

**Background:**

Information about a new coronavirus emerged in 2019 and rapidly spread around the world, gaining significant public attention and attracting negative bias. The use of stigmatizing language for the purpose of blaming sparked a debate.

**Objective:**

This study aims to identify social stigma and negative sentiment toward the blameworthy agents in social communities.

**Methods:**

We enabled a tailored text-mining platform to identify data in their natural settings by retrieving and filtering online sources, and constructed vocabularies and learning word representations from natural language processing for deductive analysis along with the research theme. The data sources comprised of ten news websites, eleven discussion forums, one social network, and two principal media sharing networks in Taiwan. A synthesis of news and social networking analytics was present from December 30, 2019, to March 31, 2020.

**Results:**

We collated over 1.07 million Chinese texts. Almost two-thirds of the texts on COVID-19 came from news services (n=683,887, 63.68%), followed by Facebook (n=297,823, 27.73%), discussion forums (n=62,119, 5.78%), and Instagram and YouTube (n=30,154, 2.81%). Our data showed that online news served as a hotbed for negativity and for driving emotional social posts. Online information regarding COVID-19 associated it with China—and a specific city within China through references to the “Wuhan pneumonia”—potentially encouraging xenophobia. The adoption of this problematic moniker had a high frequency, despite the World Health Organization guideline to avoid biased perceptions and ethnic discrimination. Social stigma is disclosed through negatively valenced responses, which are associated with the most blamed targets.

**Conclusions:**

Our sample is sufficiently representative of a community because it contains a broad range of mainstream online media. Stigmatizing language linked to the COVID-19 pandemic shows a lack of civic responsibility that encourages bias, hostility, and discrimination. Frequently used stigmatizing terms were deemed offensive, and they might have contributed to recent backlashes against China by directing blame and encouraging xenophobia. The implications ranging from health risk communication to stigma mitigation and xenophobia concerns amid the COVID-19 outbreak are emphasized. Understanding the nomenclature and biased terms employed in relation to the COVID-19 outbreak is paramount. We propose solidarity with communication professionals in combating the COVID-19 outbreak and the infodemic. Finding solutions to curb the spread of virus bias, stigma, and discrimination is imperative.

## Introduction

### Background of COVID-19 and Blaming Devices

Toward the end of 2019, a new coronavirus appeared in the city of Wuhan, Hubei Province, mainland China. On February 11, 2020, the World Health Organization (WHO) officially named the new human infectious disease “COVID-19” [1]. On March 11, 2020, it was designated as a global pandemic, spreading across 185 countries and regions [1]. The ongoing COVID-19 pandemic may have been inevitable due to the virus’s fast transmission and highly contagious nature. To date, according to the Johns Hopkins University dashboard as of August 8, 2020, there has been an overall worldwide total of 15,751,658 confirmed cases and 639,207 deaths [2]. No one could have imagined COVID-19’s rapid global spread and devastating impact.

Governments have been criticized for failing to take adequate action against COVID-19. For instance, the Chinese government was blamed for not controlling the animal trade, which was alleged to have caused the infection in humans. Early discourse contained several contributions suggesting that COVID-19 could have originated from a laboratory in Wuhan [3]. Information was spread in spite of lacking tenable scientific evidence concerning the virus’s pathology. US President Donald Trump and his administration harshly blamed China for its failure to contain COVID-19 and, by calling COVID-19 the *Chinese virus*, potentially incited racism and inadvertently attacked people of Asian descent around the world [4]. The discourse was of such low quality that a group of 27 prominent public health scientists from outside of China dismissed the biased information and pushed for a firm condemnation of misinformation and conspiracy theories about the origin and facts surrounding the virus (eg, [5]). These examples show that not only the virus but also the way it is spoken about can hurt people.

Social stigma in the context of a disease outbreak comes from an impulse to assign blame; hence, abundant research has acknowledged the social stigma and the subsequent blame and discrimination attached to COVID-19 (eg, [6-8]). The lack of a clear understanding about social stigma regarding the COVID-19 pandemic may lead to the circulation of false blame and negative bias, which jeopardizes the public’s psychosocial development and well-being. As such, it is necessary to address disease-related stigma during infectious disease outbreaks by examining stigmatizing cues and negative sentiments along with blaming information.

As of August 8, 2020, a total of 477 confirmed cases, 7 deaths, and 83,117 tested people were reported by Taiwan’s Centers for Disease Control (CDC) [9]. With the outbreak of the deadly COVID-19, Taiwan might have been in for a difficult time because of its close ties with mainland China. However, unlike many neighboring countries and regions, Taiwan has a comparatively low case-fatality rate and has not imposed a strict city lockdown. Instead, people in Taiwan have been urged to reduce their contact with others by maintaining social distancing, washing their hands frequently, and wearing face masks at all times [9]. These requests from government created many social dilemmas and violent altercations, particularly during the COVID-19 crisis. In fact, such information involved social stigma, complaining, and collective blaming often expressed through online communication to form public opinions and, in turn, affect people’s cognition. Blame is a vehicle for making meaning, through which the lay public seeks to understand unexpected risky events. Meanwhile, blaming someone is the practice of holding that agent responsible while expressing attitudes of resentment, indignation, or grievance.

### Attributing Blame and Stigma

A precondition for blame attribution is the belief that in a just world, where people behave fairly, everyone gets what they deserve [10-12]. As such, three variables including attribution of blame, responsibility, and actor intention were examined to show that the accused agents are in fact the culprits [13,14]. Previous research on blaming attribute of health risks indicates that it leads to a range of damaging social outcomes. For instance, during the COVID-19 outbreak, Chinese people living overseas experienced discrimination, and the majority of Chinese people in China exhibited discriminatory attitudes toward Chinese themselves [15]. When considering various aspects of communication in relation to public health issues, the COVID-19 pandemic tends to provoke xenophobia, discrimination, and biases with stigmatized monikers [16-18]. This shows that blaming discourse and the public’s lack of understanding of sensationalized media discourse is intricately connected to the specificities of social conditions. The source of health information for the lay public may deflect and diffuse blame with stigmatizing cues. In particular, ubiquitous online media has created an information overload that makes it difficult to differentiate between true and false information [19-21].

That certain classes of people and agents become targets for receiving blame for negative health outcomes is not a new phenomenon. For instance, placing blame on China is a familiar phenomenon and has contributed to the notion that China is an unsanitary entity. Some reporters held Chinese immigrants in New York’s Chinatown responsible for the 2013 severe acute respiratory syndrome (SARS) outbreak, despite public health officials failing to find SARS cases there [22]. The assumed health status of Chinese people led to the stigmatization of that community. Studies have emphasized that public responses and negative attitudes toward certain groups have contributed to the spread of the COVID-19 pandemic [23-25]. The naming of health risks for specific groups can fuel the negative consequences that occur as a result of the fight against infectious diseases. Therefore, researchers have confirmed the widespread collective perceptual bias against the Chinese by using stigmatizing monikers [8].

Blaming can reflect the user’s emotional state and efforts at mitigating potential losses. In particular, the use of negative tones in the background of communications can reveal the interlocutors’ intent, as they tend to come from existing sentiments of frustration and grievance [26-28]. Thus, assessing the valence of messages from news reports and commentary on COVID-19 as negative or positive can help researchers reach a basic understanding of the context.

When the public is coping with unexpected health-related risk events, stigmatizing monikers can indicate who is giving and receiving blame based on valence [29,30]. Social media has amplified the problem of stigma by spreading inaccurate and harmful information. The harm is not only medical but also includes discrimination against people at the epicenter of an outbreak [16,17,31,32]. A recent study endorsed the problem of stigma by extracting sentiment keywords from Twitter hashtags related to COVID-19 [33]. They found that the keywords “corona” and “Wuhan corona” were associated with emotions of fear and anger, while the least common emotions expressed in tweets were sadness, joy, and disgust. Perceptions of risk can be ampliﬁed or attenuated by a variety of emotions including perceived dreadfulness, lack of controllability, and unfamiliarity as projected through all types of media. Social media has proven to be one of the most influential platforms for interacting about controversial topics and making aggressive or contemptuous comments [34-38]. A positive correlation between the number of Weibo posts and the number of reported COVID-19 cases in Wuhan showed that approximately 10 additional cases were reported per 40 Weibo posts [39]. Notably, the effect size was said to be larger in Wuhan than what was observed in other cities in China.

### Goal of This Study

To summarize, this study sought to analyze how frequently online media was used to disseminate COVID-19–related information with stigmatizing cues, examine how frequently principal agents in the field are put in proximity to negative sentiment regarding the COVID-19 pandemic, and assess discourse regarding COVID-19 over time by taking into account blaming sentiment. This study draws on existing theories to understand how social stigmas and subsequent blaming present challenges, as nations grapple with restrictions on individuals’ movement and move to more normal social interaction.

### Research Questions

Four research questions (RQs) were raised to identify the interlocutor’s intent and the extent to which online media has attributed blame along with the collective expression of sentiment.

- RQ 1: How much coverage and discussion is devoted to COVID-19 and its related topics in news media and other social media sources?
- RQ 2: What stigmatizing terms are mentioned with sentiment in discussions related to COVID-19?
- RQ 3: Which targets are blamed most for the pandemic in online media?
- RQ 4: What association is there between blaming sentiment and media source in the COVID-19 pandemic?

## Methods

### Automated Computational Approach

Natural language processing (NLP) in the field of machine learning, which enables a computer to analyze, manipulate, and potentially generate human language, has been widely applied worldwide (eg, [32,35,40]). A machine learning algorithm, DivoMiner in Taiwan, with the ability to automatically identify and classify patterns in large amounts of data sets was employed. This tailored text-mining platform assisted in gaining insights from an unstructured text corpus for key terms, phrases, and sentiment assessment. The collection of various digital communication platforms was converged through automated technology, allowing for real-time aggregation, organization, and analysis of the COVID-19 discourse [17].

The timeline for this study was from December 30, 2019, to March 31, 2020, which ensured the provision of timely information related to COVID-19. It also ensured that the time periods are comparable for operational reasons such as why certain weeks may have higher demand than others or other factors that could influence blaming discourse. The research strategy included enabling DivoMiner to identify data in their natural settings by retrieving and filtering online media sources [41,42] and constructing vocabularies and learning word representations from NLP for analysis along with the research theme (stigmatizing and sentiment language) from online media genres [41-43].

### Data Collection

The leading digital news platforms in Taiwan were recruited based on their high use. At the same time, social networks and discussion forums have become increasingly important sources of news. Among all the social networking services, those with the highest penetration were YouTube and Instagram, as they reach approximately 23 million (89%) users, followed by Facebook, which has over 21 million (82%) active users, and open discussion forums, which reach over 3.3 million (95.5%) users aged between 12-38 years [41]. The aforementioned media platforms reach broad segments of the population with a daily flow of health news and discourse. In sum, the media source data recruited in this study comprised of ten mainstream news services, eleven discussion forums, one social networking service (Facebook), and two principal media sharing networks (Instagram and YouTube) for their population data set.

All publicly accessible online communications containing the target keywords posted within the 3-month timeline were automatically collected via the DivoMiner application. For example, a mainstream online newspaper, Apple Daily News, and their Facebook fan page were recruited, and the largest terminal-based bulletin board system, PTT, was observed [41,44]. However, it is worth noting that some popular social media platforms such as Twitter can serve as an accurate mirror of the population in the English-speaking world but not in the Chinese-speaking world. Hence, limiting text data to Chinese-speaking regions serves as a sufficiently effective means of gaining insight. Additionally, issues such as ethical consideration and the legality of subsequent privacy violations were less of a concern.

To ensure the efficiency of capturing opinions related to COVID-19 from unstructured text in syntactically explicit language, data were trained to include some knowledge of semantic meaning in our model [42,43,45]. To verify the feasibility and reliability of the word embedding, three coders manually labeled and checked 1500 random postings. After testing and training, the classification accuracy level reached 75% (1125/1500), which was deemed to be acceptable [41,42]. After irrelevant opinionated data were excluded (eg, shopping, nonnews), the DivoMiner classifier implemented the filtering process of the recorded data in digital form.

### Codebook Development

To parse meaning from online texts, all terms and keywords related to COVID-19 were initially collected from the official document issued by the China International Publishing Group in February 2020 [46]. To meet the requirements of computational analysis, the Word2Vec technique was employed to find continuous embedding of words. Word2Vec learns from reading 356,901 articles from the Chinese Wikipedia corpus and memorizing which words tend to appear in similar contexts [43]. After pretraining on a large corpus, it generates a multidimensional vector for each word in a vocabulary, with words of similar meaning being closer to each other. A total of 106 confirmed keywords resulted from several pilot tests creating logical terms and phrases that DivoMiner could assist in analyzing.

An example of searching a taxonomy in the tailored platform uMiner was “肺炎” (pneumonia) and the four alternative terms were “病毒性肺炎” (virus pneumonia), “冠狀病毒” (corona virus novel coronavirus), “新型冠狀肺炎” (novel coronavirus), and “2019新型冠狀病毒” (2019 novel coronavirus). The variables measuring stigmatizing monikers recruited were based on NLP for collective behavioral propensities against China or Chinese people [30,32,45]; five widely used and problematic terms with biases were “武漢病毒” (Wuhan virus), “武漢肺炎” (Wuhan pneumonia), “中國肺炎” (China pneumonia), “中國人肺炎” (Chinese pneumonia), and “中國病毒” (China virus).

DivoMiner contains a module for determining the word sentiment of each opinion. The variable measuring three sentiment tones (positive, neutral, and negative) was based on using the terms “joy,” “happy,” and “like” as points of reference for a positive tone, while “anger,” “fear,” and “sadness” were used as points of reference for a negative tone [22,25,26]. The synonyms of emotion words and adjectives not associated with the aforementioned emotions were labelled as having a neutral tone [25-27,30,43]. Negative sentiments are ideal indicators of collective perceptual bias in measuring the blaming of accused culprits [14,19-23]. A randomly assigned group of the 1200 sample posts were cross-checked by the first author and two trained research assistants. Disagreements were resolved by discussion for reaching consensus. An acceptable level of agreement of 77% (924/1200) was reached at the end. The interrater reliability employed the Cohen kappa coefficient by computing sentiment and targeted figures, groups, and organizations for the online postings. The interrater reliability was 0.5901 (95% CI 0.49-0.71; *P*=.06) and rated as moderate [42,43].

## Results

### Frequency and Trend of COVID-19 Mentions

We identified a total of 1,073,983 texts about COVID-19 from 24 online sources in Taiwan over 3 months. The first text on COVID-19 was a news article published on HiNet on December 31, 2019. It reported that Taiwan’s CDC had started in-flight disinfection and quarantine measures in response to the pneumonia epidemic in Wuhan. Notably, COVID-19 was confirmed to have spread to Taiwan 3 weeks later.

Almost two-thirds of the 1,073,983 texts on COVID-19 came from news services (n=683,887, 63.68%), followed by Facebook (n=297,823, 27.73%), discussion forums (n=62,119, 5.78%), and Instagram and YouTube (n=30,154, 2.81%). The average daily volume of COVID-19 texts was 7354 (range 0-14,561) from news services, 3202 (range 0-5937) from Facebook, 324 (0-678) posts on Instagram and YouTube, and 668 (range 0-1264) on discussion forums.

**Figure 1:**
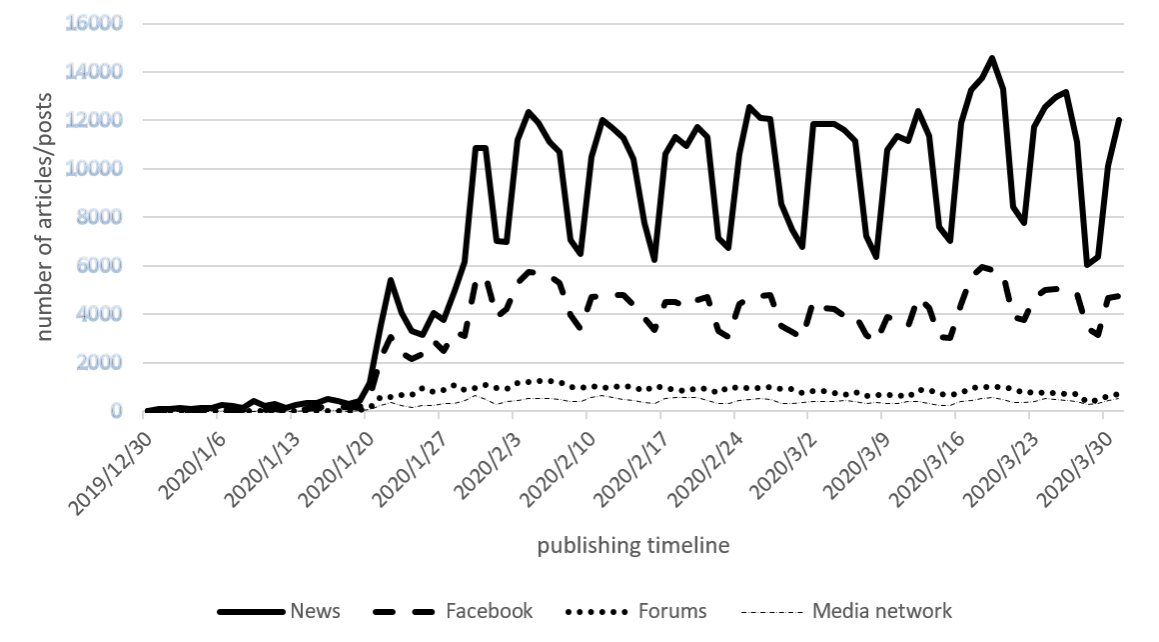
The intensity of communication about COVID-19 in social media and news services.

The amount of COVID-19 coverage and discourse shows several noteworthy patterns. First, until January 19, 2020, the daily number of new stories was always considerably less than 500 but that number rose to more than 1000 on January 20. The news stories exploded on January 21, 2020, with approximately 3500 stories and slightly less than 5500 the day after. From then on, the number of news stories sourced by news services rose to about 12,000 on weekdays and between 6000 and 8000 on weekends. Second, the all-time high was reached in the week beginning on March 16. Four of the five weekdays in that week were days when the intensity of coverage was the greatest over the 3-month time frame. Third, there was a striking weekly rhythm in the time frame, with the number of stories falling from approximately 12,000 stories per day on weekdays to between 6000 and 8000 on weekends. Fourth, the pattern for the other sources by and large mirrors the development of the news services output. Figure 1 provides an overview of the developments.

During infectious disease outbreaks, the underlying mechanism of social media posts connecting users’ risk perceptions was observed to be frequently high, with resulting cathartic effects. The keyword analysis identified two key phrases, “Tsai Ing-wen” (n=1,082,632; Taiwan’s president) and “pneumonia” (n=1,008,486), which received the highest total frequency, followed by the geographical nomenclature “Wuhan” (n=762,004) and “China” (n=343,489). Masks and mask-rationing plans were the fifth most frequently used phrase (n=305,769). Two targets linked to COVID-19 followed suit: ethnic groups of new immigrants (eg, mainland Chinese, Vietnamese, Filipinos, and Indonesians; n=7299) and Dr Li Wenliang, an ophthalmologist who attempted to alert government and the public to the imminent danger in the early phases of the pandemic (n=6004). The results showing a total of 51 frequent Chinese keywords with English translations is attached in Multimedia Appendix 1. A descriptive analysis of the top 20 high-frequency words in descending order is presented in Table 1.

To investigate the reactions pertaining to each theme, six themes including disease; infection prevention; geographical naming; organizations, institutes, and events; policy; and political figures were frequently covered. In comparison, the two themes that were least covered across various sources over time were those concerning nonpolitical figures and groups and occupations. The mean score gives the measurement of the central tendency for the analysis of thematic data. The descriptive analysis of the attributes of COVID-19 messages are presented in Multimedia Appendix 2 as the total amount of discussion, means, SD, and ranges of key terms assessed.

**Table 1:** High-frequency words related to COVID-19 in Taiwan’s online communication.

COVID-19–related words	Frequency, n
Tsai Ing-Wen (or Taiwan political figure or Taiwan President)	1,082,632
Pneumonia	1,008,486
Wuhan	762,004
Wuhan pneumonia	715,719
China	343,489
Mask or mask-rationing plan	305,769
Coronavirus	261,643
Confirmed case	185,818
Disinfection	99,817
Wash hands often/carefully	96,593
Mandatory quarantine, self-monitored quarantine	91,097
WHO^a^	81,134
Taiwan comrade (or cross-strait charter for Taiwan businessman in China)	69,310
Chen Shih-Chung (or Minister of Health and Welfare)	62,870
City lockdown	51,261
Soo Tsing Tshiong, Prime Minister of Taiwan, Executive Yuan	50,863
Cruise, Westerdam, Aquarius, World Dream, or Diamond Princess	46,906
Xi Jingping or leader of communist party, Chair Xi, or Chair	40,664
Vaccine	29,559
Suspected case	23,926

^a^WHO: World Health Organization.

### COVID-19 and Stigmatizing Cues

The usage of “pneumonia” and “virus” can be traced to the day of December 30, 2019. Thus, the stigmatizing term with negative sentiment in discussions related to COVID-19 appeared on the next day. With increasing interest in misusing the term “Wuhan pneumonia” (n=639,456), it comprised approximately one-third of overall media sources. A chi-square test of independence showed that there was a significant association between keyword usage and media platforms (χ^2^_9_=2,311,455, *P*<.001). The stigmatizing terms were presented most frequently in the news (n=519,261, 37.0%), followed by Facebook (n=193,249, 34.4%), forums (n=38,650, 36.2%) and other social media networks (Instagram and YouTube; n=19,325, 32.1%). Table 2 presents a comparison of usage between stigmatized and nonstigmatized terms adopted in COVID-19 discussions on different media platforms.

**Table 2:** Comparison of nonstigma (recommended) keywords and stigmatizing terms by media type.

Variables	Nonstigmatized, n (%)	Stigmatized, n (%)
News (n=1,405,306)	886,045 (63.0)	519,261 (37.0)
Facebook (n=561,298)	368,049 (65.6)	193,249 (34.4)
Forums (n=106,807)	68,157 (63.8)	38,650 (36.2)
Instagram and YouTube (n=60,219)	40,894 (67.9)	19,325 (32.1)
Total (n=2,133,630)	1,363,145 (63.9)	770,485 (36.1)

For the stigmatizing terms with a sentiment assessment, the terms “Wuhan pneumonia” and “China virus,” as potentially offensive terms, accumulated a total of 631,192 posts with associated sentiments. A negative tone of more than 50% was associated with blame (n=331,550, 52.3%), compared to a positive tone of 26.86% (n=169,541) and a neutral tone of 20.61% (n=130,101). There was a significant relationship between sentiment and stigmatizing terms (χ^2^_9_=994,650, *P*<.001). Table 3 presents a comparison of sentiments associated with stigmatizing terms on different media platforms.

**Table 3:** Sentiment assessment of stigmatizing terms by media type.

Variables	Positive, n (%)	Neutral, n (%)	Negative, n (%)
News (n=359,574)	106,091 (29.5)	85,681 (23.8)	167,802 (46.7)
Facebook (n=207,768)	49,150 (23.7)	29,084 (14.0)	129,534 (62.3)
Forum (n=43,464)	6866 (15.8)	11,904 (27.4)	24,694 (56.8)
Instagram and YouTube (n=20,386)	7434 (36.5)	3432 (16.8)	9520 (46.7)
Total (n=631,192)	169,541 (26.9)	130,101 (20.6)	331,550 (52.5)

### Most Blamed Targets

The targets blamed most often were four leading political figures (Soo Tsing Tshiong, Chen Shih-Chung, Tsai Ing-wen, and Xi Jinping), a group of immigrants to Taiwan, and Dr Li Wenliang in China. The number of sentiment assessments ranged from a minimum of 6004 to a maximum of 935,691. In comparison, the least posts and coverage concerning sentiment were on policy, treatment, and welfare organizations (ranging from 0 to 1328). Quantifying and understanding the development of comments with sentiment in news services and social media activity revealed that a politician, Soo Tsing Tshiong (the Taiwanese Prime Minister), drew the most attention with polarized tones, both negative (n=456,187, 48.8%) and positive (n=280,814, 30.0%). Chen Shih-Chung, the Taiwan Minister of Health and Welfare, followed with more negative (n=22,033, 42.0%) than positive tones (n=15,432, 29.4%), as he oversaw resources across ministries and private stakeholders to fight against COVID-19. Additionally, Xi Jinping, the President of the People’s Republic of China (PRC), and issues related to him were associated with more negative (n=8148, 54.6%) than positive tones (n=2859, 19.1%).

Sentiment analysis alongside nonpolitical targets such as new immigrants, foreign labor, and foreign spouses connected more negative (n=3153, 43.2%) than positive emotions (n=2105, 28.8%). Unexpectedly, Li Wenliang, the whistleblower, and issues related to him were associated overwhelmingly with negative (n=4358, 72.6%) than positive discussions (n=390, 6.5%). Dr Li who brought the problem about the impending virus to others’ awareness and issues related to him were not praised in the early months of the virus outbreak. Instead, Dr Li and issues related to him became a target of critics. The only exception was Tsai Ing-wen, President of Taiwan, with more positive comments (n=7786, 45.7%) than negative comments (n=3696, 21.7%) across media. Specifically, the positive comments related with her were mainly in news services (n=5318, 58.0%), compared with social media: Facebook (n=2714, 51.6%), forums (n=704, 39.1%), and YouTube and Instagram (n=252, 34.6%). The sentiment used to discuss this political leader differed considerably across media types (χ^2^_9_=11,088 *P*<.001).

Targeting the top six figures and groups, a 3 x 4 analysis of variance, with three sentiment tones (positive, neutral, and negative) and four media platforms (news, Facebook, discussion forums, and Instagram and YouTube) as between-subjects factors, revealed the main effects of tone (*F*_2,60_=1.13, *P*=.33) and media platform (*F*_1,60_=11.90, *P*=<.001). Hence, post hoc comparisons using the Tukey honestly significant difference test showed a statistically significant difference between the three different media platforms (*P*=.003): (1) news and Facebook, (2) news and forums, and (3) news and social networks (Instagram and YouTube). The coverage of online news with sentiment tone showed a significantly higher average and SD (mean 4117, SD 3799) than the Facebook posts (mean 1778, SD 1816). The effect sizes for these two significant effects (news and Facebook, and news and forums) were 0.79 and 1.33, respectively. Additionally, sentiment expressed on online forum posts had a significantly higher average score of 530 (SD 346), compared to Instagram and YouTube (average score 225, SD 217), with an effect size of 1.45. Taken together, these results suggest that news coverage with a sentiment tone had an effect on Facebook, forum posts, and Instagram and YouTube. Specifically, our results suggest that when news articles involved emotions, the sentiment carries over to social media. The means and SD for the factorial design are presented in Table 4.

**Table 4:** Differences between media venue with sentiment on targets according to post hoc tests.

Media^a^	Mean (SD)	Mean differences			
		News	Facebook	Forums	Instagram and YouTube
News	4177 (3799)	—^b^	—	—	—
Facebook	1778 (1816)	2339 (0.79)^c,d^	—	—	—
Forums	530 (355)	3587 (1.33)^c,e^	12,482	—	—
Instagram and YouTube	225 (217)	3892 (1.45)^c,e^	1553	305	—

^a^Cell size n=18.

^b^Not applicable.

^c^Effect sizes are indicated in parentheses.

^d^*P*=.002.

^e^*P*<.001.

## Discussion

### Principal Results

This study uncovered a pattern of how the online blame for the COVID-19 pandemic was directed at groups and figures whose influential users had pre-existing grievances with or frustrations about. People’s practices of ascribing blame remain fallible, and it seems natural to assume that their perceptual biases are also features of the object of such descriptions and not only features of their very practice. Using social media to follow news about COVID-19 and related topics compensated for traditional news in terms of gathering a diverse and broad variety of general health news. The public develops interpretations of COVID-19 through a variety of resources, most notably representations presented by mainstream online news. Overall, there was a strong and positive correlation between the negative and positive tones of media sources. Increases in discussion frequency were correlated with increases in sentiment tones on online media.

Stigma is disclosed through negatively valenced responses rather than positive ones associated with figures related to COVID-19. For instance, Soo Tsing Tshiong, Taiwan’s Prime Minister, was associated with the most blame, despite that Soo is considered to be one of the local figures responsible for prevention and policy implementation. The blame connecting with him appeared mostly in the news (n=519,261, 67.4%), followed by Facebook (n=193,249, 25.1%) and discussion forums (n=38,650, 5.0%). Notably, Xi Jinping, the PRC President, and issues related to him were associated with more negative tone comments from the news (n=4223, 51.8%), followed by Facebook (n=2672, 32.8%) and discussion forums (n=893, 11.0%). The accused agents are in fact the culprits who are associated frequently with negative sentiment.

Taiwan’s news media has been described as professional and independent [42]; however, online news services have been facilitating a negative tone. Online news has persistently used stigmatizing terms to initiate the adoption of stigma-related emotion words. In this study, they appeared first in the news and showed a resurgence of use in the week of January 20, 2020; afterwards, they appeared on Facebook and online forums. Online news is a hotbed of negativity and drives negative sentiment and blame in other media. The stigmatizing terms were clearly deemed offensive, and they might have contributed to recent backlashes against China and Chinese people by encouraging and directing blame [6-8]. Understanding the nomenclature and biased terms employed in relation to the COVID-19 outbreak is paramount while considering the online public’s responses and feelings around making biased judgments. Stigmatizing language linked to the pandemic used by online media influencers shows a lack of civic responsibility, encouraging bias and hostility.

### Limitations

Despite this study’s attempts to establish the accuracy of the inferred meaning from all media texts, this study has several limitations related to research design and analytical workflow. First, the online media data set was characterized by a diversity in genres, which did not use fine-grained information. In particular, the data derived from individual social media accounts came in the form of sparse and short texts, which were less likely to lead to insights into the identification of ambiguous information. Working with data from social media remains subjective, and it is challenging to quantify synthesized data that do not necessarily have a closer common claim on objective truth. Second, our automated methods inevitably fell short in reducing a text to a model that encapsulated all important Chinese sentiment lexicons by training sentiment analysis models [43]. In this case, the neutral tag is one of the most important parts of the research problem. Despite preprocessing and postprocessing data being applied to capture the bits of context, tagging criteria should be more consistent. In addition, it should be noted that more efficient blend words or tags with shorter orthographic and phonetic length were often used in social media, compared to the accompanying key terms with formal spelling in traditional news. This difference indicated that data mining based on keyword matching could underestimate actual volume of usage. Last, the current assessment of sentiment tones requires a deeper understanding of frequent user’s affective expressions from online media [26]. The classifiers of sentiment assessment might be more precisely generated for measurement by considering the multifaceted nature of Chinese keywords in various media.

### Comparison With Prior Work

The WHO and global medical authorities have agreed to veer away from naming illnesses after places or groups of people because using such names could lead to collective perceptual bias, stigma, and inaccurate assumptions [5]. However, consistent with previous studies (eg, [7,8]), the adoption of the problematic moniker “Wuhan pneumonia” had a high frequency of collective production and consumption. Our data also showed the high association of COVID-19 with China and a specific city within China through references like “Wuhan pneumonia,” potentially encouraging xenophobia. Comparing the results with earlier findings (eg, [18,38]), news and social networks were observed to be rough proximations of beliefs providing georeferenced sentiment connecting the virtual and material spaces of a health crisis. Additionally, Tsai Ing-wen has been associated with more favorable affective responses than others, while Chen Shih-Chung was later observed to connect to nonblameworthy statements amid the COVID-19 flare-up at the end of March 2020. The xenophobia that spread during the COVID-19 outbreak showed that individuals can enhance the media’s exposure and credibility, and consequently, shape the public’s views and appraisals. The positive mood toward the two Taiwanese politicians offers an opportunity to reflect on the lessons learned in this pandemic’s framing of online heroization dynamics [23].

### Conclusions

This study explores the mechanisms of how blame was associated with various targets in online communications during the COVID-19 outbreak in a widely used language other than English. Given the impact of the online discourse about COVID-19 to date, it is crucial to reduce stigma amid the pandemic. This timely report can be used to inform policies and to stimulate research related to how societies deal with pandemics with stigma mitigation. Particularly, sentiment analysis has great potential in tracing sources for predicting the spread of infectious diseases with emotions [26,29,38,45]. Online data sources such as mobile phones can help researchers discover new pathogens at the community level and can be used to leverage big data and intelligent analytics for public health [30].

This study investigating the contentious and distorted nature of online media dynamics concludes that the collective behavior of perceptual bias against COVID-19 existed in daily communications among Taiwanese users. At the local scale, social media users broadly occupy the same geographical turf, which is why it is considered appropriate to explore the pandemic as a reference for future study. Media users have fueled the unprecedented dissemination of stigmatizing terms with negative tones to direct hostility and blame. Because of this, harmful language can have higher stakes, and the risk of offline harm can become exacerbated. Thus, the awareness of blaming devices is promoted through empowering individuals, health communication researchers, health care professionals, and policy makers to take responsibility for their actions. We propose solidarity with communication professionals in combating the COVID-19 outbreak and for finding solutions to curb the spread of virus bias, stigma, and discrimination.

## Appendix

Multimedia Appendix 1 Categorized keywords in Chinese and translation in English.

Multimedia Appendix 2 Topic related to COVID-19 online communication in Taiwan.

## Abbreviations

CDC: Centers for Disease Control
NLP: natural language processing
PRC: People's Republic of China
RQ: research question
SARS: severe acute respiratory syndrome
WHO: World Health Organization
