# Supplementary material for: Communicative Blame in Online Communication of the COVID-19 Pandemic: Computational Approach of Stigmatizing Cues and Negative Sentiment Gauged With Automated Analytic Techniques
Source: J Med Internet Res. 2020 Nov 25;22(11):e21504. doi: 10.2196/21504 (PMC7690967; doi:10.2196/21504)
Supplement: Multimedia Appendix 1 [file jmir_v22i11e21504_app1.docx]

**Multimedia Appendix 1**

Appendix I. Categorized keywords in Chinese and translation in English

**Theme 1.疫病名稱 Diseases**

1. 肺炎 pneumonia
2. 冠狀病毒coronavirus
3. 新型冠狀/新型冠狀病毒/nCoV novel coronavirus/nCoV
4. 2019新型冠狀病毒 2019 novel coronavirus/2019-nCoV
5. 病毒性肺炎virus pneumonia
6. 中國病毒China virus
7. 中國人肺炎Chinese virus
8. 中國肺炎China pneumonia
9. 武漢病毒Wuhan virus
10. 武漢肺炎Wuhan pneumonia

**Theme 2.傳染防控 Infection Prevention**

1. 人傳人 person-to-person/human-to-human transmission
2. 潛伏期 incubation/latent period
3. 無症狀 silent/asymptomatic period
4. 飛沫傳播 droplet transmission
5. 接觸傳播 contact transmission
6. 醫院/院內感染 nosocomial infection; hospital-acquired infection
7. 確診病例 confirmed case
8. 疑似病例 suspected case
9. 發病率 incidence rate
10. 死亡率 mortality rate

**Theme 3. 政策舉措 Policy**

1. 遏制疫情蔓延 (contain the outbreak)
2. 封城 (city lockdown)
3. 延遲開學 (postpone reopening of schools)
4. 居家檢疫 (自我隔離) (quarantine yourself in your home/self-monitored quarantine)
5. 疫苗 (vaccine)
6. 口罩/口罩實名制 (face mask/mask-rationing plan)
7. 洗手/勤洗手 (wash hands often/carefully)
8. 消毒 (disinfection)
9. 避免去人多的地方 (avoid crowds)

**4.地理 Geographical naming**

1. 中國 (China)
2. 湖北/湖北省 (Hubei/Hubei province)
3. 武漢 (Wuhan)
4. 台灣 (Taiwan)

**5. 機構、 黨派、事件Organizations, Institutes, & Events**

1. 世衛組織/世衛/WHO (WHO/World Health Organization)
2. 台灣疾病預防控制中心 (Taiwan CDC/Center for Disease Control and Prevention)
3. 共產黨/中共/阿共 (Communist Party/CCP)
4. 郵輪 (遊輪 or 威斯特丹or 寶瓶星or 世界夢or鑽石公主) (Cruise liners/Cruise ship or Westerdam or Aquarius or World Dream or Diamond Princess)

**6.群體、職業名稱 Groups & Occupations**

1. 臺胞/台商 (Taiwanese comrade/businessmen)
2. 包機/台商包機 (cross-strait charter for Taiwan businesspeople in mainland China)
3. 醫療/醫護/健康照護人員 (medical personnel/nursing professionals/healthcare workers)
4. 新住/外勞/外配 (new immigrants/foreign labor/foreign spouse)

**7.政治人物political figures**

1. 大陸政治人物 (Mainland China politicians as a whole)
2. 習近平/中共領導/習主席/主席 (Xi, Jingpin/ leader of China Communist Party/Chair Xi/Chairman)
3. 台灣政治人物 (Taiwan politicians as a whole)
4. 蔡英文/總統 (Tsai, Ing-wen/President)
5. 蘇貞昌/蘇院長/行政院長/行政院) (Soo Tsing Tshiong/Soo Premier/Prime Minister/head of Executive Yuan/ Executive Yuan)
6. 陳時中/衛福部部長/部長/衛福部長/疾病管制局 (Chen Shih-Chung/ Minister of Health and Welfare/Centers for Diseases Control and Preventions (CDC)/ Minister of CDC/center of Epidemic Command Center)
7. 陳明通/大陸委員會/陸委會) (Chen Ming-Tong/Minister of Mainland Affairs Council)

**8. 非政治人物non-political figures**

1. 李文亮/李醫師 (Li wenlian/Dr. Li)
2. 鐘南山/抗煞英雄/抗煞專家 (Zhong Nanshan/expert in respiratory diseases/a hero of the fight against SARS)
3. 藝人/小S/徐熙娣/大S/范瑋琪/范范/范建/黑範) (Names of local celebrities/actress/host of variety shows)
