# Supplementary material for: Communicative Blame in Online Communication of the COVID-19 Pandemic: Computational Approach of Stigmatizing Cues and Negative Sentiment Gauged With Automated Analytic Techniques
Source: J Med Internet Res. 2020 Nov 25;22(11):e21504. doi: 10.2196/21504 (PMC7690967; doi:10.2196/21504)
Supplement: Multimedia Appendix 2 [file jmir_v22i11e21504_app2.docx]

**Multimedia Appendix 2**

Appendix II. Topic related to COVID-19 online communication in Taiwan

| Topic related to COVID-19 | Total | Mean | SD | Min. | Max. |
| --- | --- | --- | --- | --- | --- |
| Virus & related  diseases | 2031898 | 290271 | 410028 | 2477 | 1008486 |
| Political figures | 1245515 | 207586 | 429367 | 1976 | 1082632 |
| Geographical naming | 1176887 | 392296 | 347882 | 71394 | 762004 |
| Policy | 686550 | 76283 | 95095 | 228 | 305769 |
| Infection prevention | 292456 | 29246 | 55415 | 621 | 185818 |
| Org., Institutes & Events | 152213 | 38053 | 33304 | 7359 | 81134 |
| Groups & occupations | 91540 | 30513 | 33729 | 8154 | 69310 |
| Non-political figures | 14697 | 5899 | 3096 | 1324 | 6717 |
